# Supplementary figures and images for: A novel steganography method for binary and color halftone images
Source: PeerJ Comput Sci. 2022 Aug 16;8:e1062. doi: 10.7717/peerj-cs.1062 (PMC9455272; doi:10.7717/peerj-cs.1062)

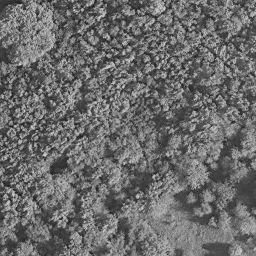

Supplement: Supplemental Information 1 [file peerj-cs-08-1062-s001.zip › peerj_matlab/cover_imgs/forest22_256gray.png]

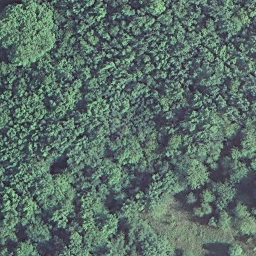

Supplement: Supplemental Information 1 [file peerj-cs-08-1062-s001.zip › peerj_matlab/cover_imgs/forest22_256rgb.png]

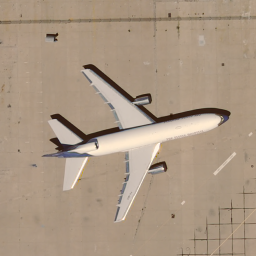

Supplement: Supplemental Information 1 [file peerj-cs-08-1062-s001.zip › peerj_matlab/cover_imgs/airplane80_256rgb.png]

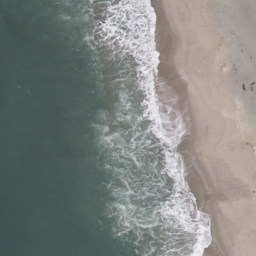

Supplement: Supplemental Information 1 [file peerj-cs-08-1062-s001.zip › peerj_matlab/cover_imgs/beach09_256rgb.png]

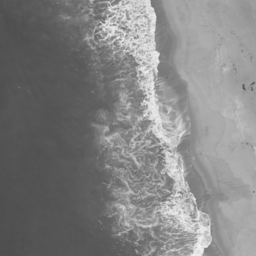

Supplement: Supplemental Information 1 [file peerj-cs-08-1062-s001.zip › peerj_matlab/cover_imgs/beach09_256gray.png]

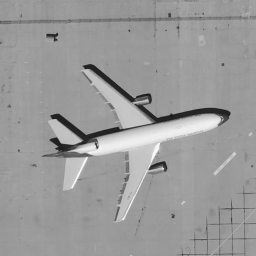

Supplement: Supplemental Information 1 [file peerj-cs-08-1062-s001.zip › peerj_matlab/cover_imgs/airplane80_256gray.png]
